# Laparoscopic Resection of a Large Jejunal Diverticulum-Like Gastrointestinal Stromal Tumor: A Case Report

**DOI:** 10.70352/scrj.cr.25-0550

**Published:** 2026-01-27

**Authors:** Ryosuke Mizuno, Shintaro Okumura, Shinya Otsuki, Shigeo Hisamori, Shoichi Kitano, Yoshiyuki Kiyasu, Ryuhei Aoyama, Yu Yoshida, Takehito Yamamoto, Masahiro Maeda, Masazumi Sakaguchi, Takashi Sakamoto, Keiko Kasahara, Nobuaki Hoshino, Ryosuke Okamura, Yoshiro Itatani, Shigeru Tsunoda, Koya Hida, Kazutaka Obama

**Affiliations:** 1 Department of Surgery, Kyoto University Graduate School of Medicine, Kyoto, Kyoto, Japan; 2 Department of Diagnostic Pathology, Medical Research Institute Kitano Hospital, PIF Tazuke-Kofukai, Osaka, Osaka, Japan

**Keywords:** emergency surgery, gastrointestinal stromal tumors, jejunal diverticulum, laparoscopic surgery, minimally invasive surgery, perforation

## Abstract

**INTRODUCTION:**

Gastrointestinal stromal tumors (GISTs) that present as large jejunal diverticulum-like lesions are exceedingly rare, with only 8 cases reported in the English literature to date. Notably, all previously documented cases were treated via open surgery. To our knowledge, this is the first report of successful laparoscopic resection of such a lesion. This case contributes novel insight into the management of rare GIST presentations and demonstrates the potential applicability of minimally invasive surgery.

**CASE PRESENTATION:**

A man in his 70s was incidentally diagnosed with a large jejunal diverticulum-like structure on abdominal CT. The lesion eventually perforated during follow-up, requiring emergency laparoscopic partial jejunal resection. Due to severe inflammation and infiltration around the lesion, partial colectomy was also required. Intracorporeal anastomoses were performed for both the jejuno-jejunal and colo-colic reconstructions. Histopathological analysis confirmed the diagnosis of GIST, revealing a 10.2-cm mass that had completely replaced the original jejunal wall structure. The tumor was thought to have arisen from the jejunal muscularis propria and expanded outward, creating a pseudo-diverticular appearance. Postoperative recovery was uneventful; however, multiple liver metastases developed shortly thereafter. The patient was started on imatinib therapy, resulting in a sustained reduction in tumor size.

**CONCLUSIONS:**

This case demonstrates that laparoscopic resection is feasible even for rare and complicated presentations of jejunal GISTs, such as those mimicking large jejunal diverticula. It provides new evidence supporting the safety and effectiveness of minimally invasive surgery in emergency settings involving tumor perforation.

## INTRODUCTION

Gastrointestinal stromal tumors (GISTs) are the most common mesenchymal tumors of the gastrointestinal tract. While GISTs frequently arise in the stomach, approximately 20% originate in the small intestine.^1)^ Among these, GISTs presenting as jejunal diverticulum-like lesions are extremely rare, with only 8 cases reported in the English literature.^2)^ Moreover, the development of jejunal diverticulum-like GISTs is unclear, and it is often difficult to determine their true origin.^3)^

Laparoscopic resection has been widely adopted for the treatment of GISTs.^4)^ However, jejunal diverticulum-like GISTs are often large and perforated at the time of diagnosis.^5)^ Consequently, all previously reported cases were managed with open surgery.^6)^ Here, we report a case of a large jejunal diverticulum-like GIST that was safely resected laparoscopically.

## CASE PRESENTATION

**Fig. 1:**
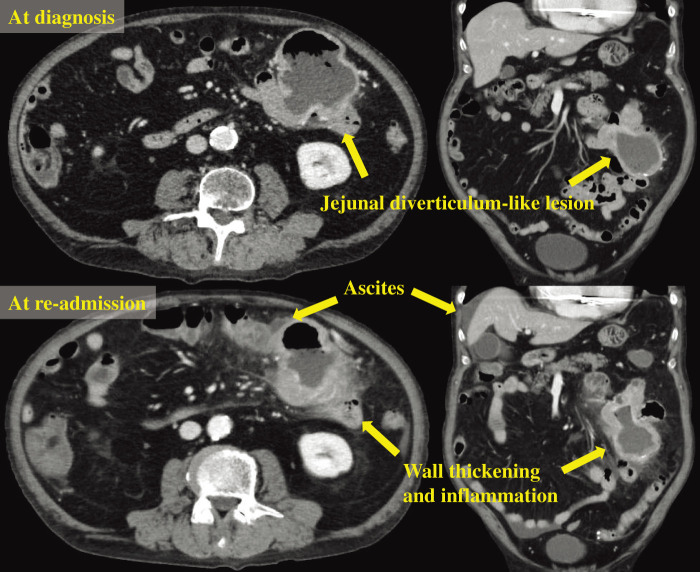
Preoperative CT imaging. A contrast-enhanced CT scan incidentally revealed a small intestinal mass. At readmission, CT demonstrated wall thickening around the jejunal diverticulum-like lesion and the development of ascites.

The patient was a man in his 70s with a BMI of 22.7 kg/m^2^ and a medical history of cardiac sarcoidosis and complete atrioventricular block. A contrast-enhanced CT scan incidentally revealed a small intestinal mass (**Fig. 1**). Double-balloon endoscopy was performed, revealing a diverticulum-like structure with ulceration located just distal to the ligament of Treitz. Biopsy of the ulcer showed no evidence of malignancy. However, 5 days after endoscopy, the patient was readmitted with hematochezia and anemia. CT demonstrated wall thickening around the jejunal diverticulum-like lesion and the development of ascites (**Fig. 1**). Although he did not have a fever and his other vital signs were stable, inflammatory markers were elevated (white blood cell count [WBC], 14210/μL; C-reactive protein [CRP], 24.6 mg/dL), and localized peritoneal irritation was observed. The patient was diagnosed with peritonitis due to jejunal perforation, and emergency laparoscopic surgery was performed. His vital signs were stable, and because he was taking systemic steroids—with an associated increased risk of surgical site infection—we elected to proceed with a laparoscopic approach.

**Fig. 2:**
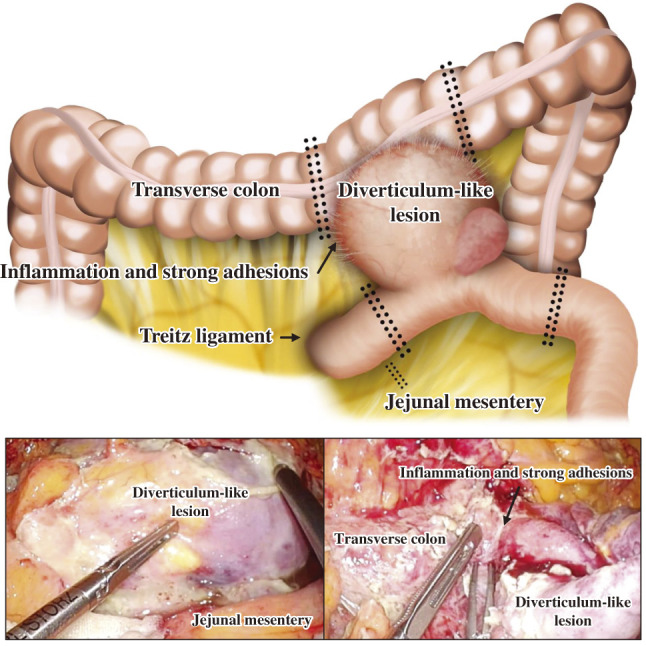
Intraoperative view of tumor. A large jejunal diverticulum-like lesion was observed with severe inflammation and strong adhesions between the lesion and the transverse colon.

**Fig. 3:**
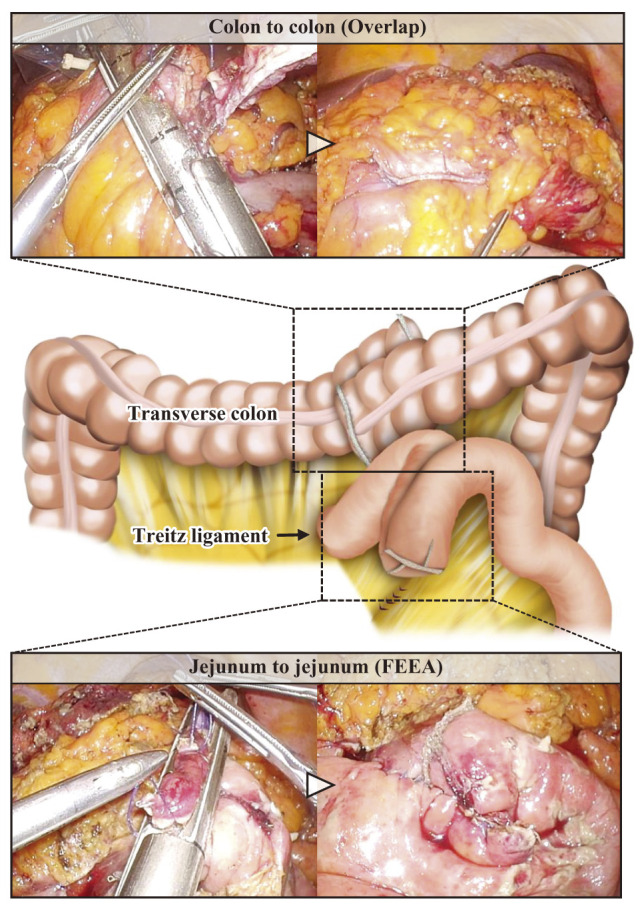
Intraoperative view after anastomosis. Intracorporeal anastomosis was performed using functional end-to-end anastomosis (FEEA) for the jejunum and overlap anastomosis for the transverse colon, respectively.

Five ports were placed in the split-leg position (umbilical, 12 mm; bilateral flank, 5 mm; and bilateral lower abdomen, 12 mm). Intestinal fluid-like ascites was diffusely present throughout the abdominal cavity. A large jejunal diverticulum-like lesion was covered by the omentum and small intestine, which were carefully dissected to expose the lesion. Severe inflammation and strong adhesions were observed between the diverticulum-like lesion and the transverse colon (**Fig. 2**). A mass-like lesion was observed to be lodged between the diverticulum and the transverse colon. Considering the possibility of a malignant tumor, the jejunum was transected between the beginning of the jejunum and the anal side of the lesion, with combined resection of the transverse colon to secure a safety margin. Intracorporeal anastomosis was performed using functional end-to-end anastomosis for the jejunum and overlap anastomosis for the transverse colon, respectively (**Fig. 3**). To prevent anastomotic leakage, additional reinforcing sutures were placed laparoscopically at the crotch of the anastomosis. The patient had no significant history of atherosclerotic disease, and intraoperative perfusion assessment with indocyanine green (ICG) was not performed. To avoid tumor seeding, the specimen was placed in a retrieval bag and extracted through the umbilical incision, which was extended by 5 cm. Finally, the abdominal cavity was irrigated with 6 L of normal saline, and drains were placed in the bilateral subphrenic spaces and the pelvic cavity. The operative time was 317 min, with a blood loss of 310 mL, and 2 units of red blood cells were transfused intraoperatively.

**Fig. 4:**
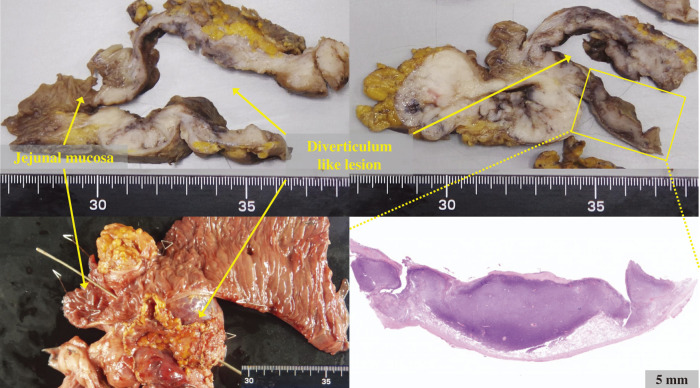
Histopathological analysis. Histopathological examination revealed that the intestinal wall of the 10.2-cm jejunal diverticulum-like lesion was entirely replaced by tumor tissue, with no remaining mucosal architecture.

Histopathological examination revealed that the intestinal wall of the jejunal diverticulum-like lesion was entirely replaced by tumor tissue, with no remaining mucosal architecture; perforation was observed at the apex of the diverticulum-like structure (**Fig. 4**). Subserosal invasion of the transverse colon was observed. Immunohistochemistry results were positive for CD117 and DOG1. The Ki-67 index was 4.6%, the mitotic index was 2 per 50 high-power field (HPF), and the maximum tumor diameter was 10.2 cm. Based on the modified Fletcher risk classification, the tumor was diagnosed as high-risk GIST. The surgical margins were negative.

The patient was admitted to the ICU postoperatively; however, the clinical course was favorable, and he was transferred to the general ward on the following day. The nasogastric tube was removed on POD 3. Postoperative inflammation was prolonged but gradually improved with antibiotic therapy consisting of meropenem, teicoplanin, and micafungin. Oral intake was resumed on POD 11, all drains were sequentially removed by POD 12, and the patient was discharged on POD 22, with no remarkable complications. Because the patient had cardiac sarcoidosis, adjuvant imatinib therapy was not administered. At 9 months postoperatively, follow-up CT revealed 4 hepatic metastases, each measuring up to 1 cm. Imatinib was introduced with careful monitoring of cardiac function. All hepatic metastases became difficult to identify on CT, and the patient remained alive 18 months postoperatively.

## DISCUSSION

In this report, we present a case of a perforated large GIST with a diverticulum-like appearance in the jejunum that was successfully resected laparoscopically. To the best of our knowledge, this is the first documented case of laparoscopic resection of such lesion (**Table 1**).

**Table 1:** Cases of reported gastrointestinal tumors in jejunal diverticula

Author (year)	Age, sex	Perforation	Size	Location	Surgical approach	Risk	Imatinib	Prognosis
Schepers and Vanwyck (2009)^12)^	56, Female	N.A.	N.A.	N.A.	Open	N.A.	N.A.	Follow-up information N.A.
Sadaf (2010)^13)^	22, Female	No	4.0 × 4.0 cm	N.A.	Open	Low	No	Well at 9 months post-surgery
Jaykar (2013)^14)^	58, Male	Yes	3.0 × 2.0 cm	15–20 cm from Treitz ligament	Open	Low	N.A.	Follow-up information N.A.
Shoji (2014)^10)^	61, Male	Yes	5.9 × 5.3 cm	40 cm from Treitz ligament	Open	Moderate	Yes	Follow-up information N.A.
Petroianu (2016)^15)^	59, Male	Yes	8.0 × 4.5 cm	80 cm from Treitz ligament	Open	N.A.	Yes	Recurrence at 1-year; resected; well at 38 months
Arata (2020)^5)^	46, Male	Yes	7.0 × 6.5 cm	N.A.	Open	High	Yes	No recurrence (duration N.A.)
Chung (2021)^16)^	69, Female	Yes	5.5 × 4.0 cm	30 cm from Treitz ligament	Open	N.A.	No	Well at 6 weeks post-surgery
Chiew (2023)^2)^	48, Female	No	5.0 × 5.0 cm	50 cm from Treitz ligament	Open	Moderate	No	Well at 8 months post-surgery
Present case	73, Male	Yes	10.2 × 6.0 cm	Just distal to Treitz ligament	Laparoscopic surgery	High	Yes	Liver recurrence at 9 months; well at 18 months post-surgery

N.A., not available

Small intestinal GISTs often lack specific symptoms and are frequently diagnosed late, resulting in tumors that are typically large at the time of diagnosis, with approximately two-thirds measuring more than 5 cm in diameter.^5)^ Additionally, they are more likely to follow a malignant course than gastric GISTs.^6)^ Diverticulum-like jejunal GISTs are particularly rare and prone to delayed diagnosis. In previous reports, 63% of such cases (5 of 8) involved tumor perforation, with tumors exceeding 5 cm at the time of diagnosis.^1)^ In the present case, the tumor had also perforated, and the resected specimen measured over 10 cm in diameter. Perforation occurred shortly after the endoscopic biopsy procedure; however, the perforation site was located at the apex of the diverticulum-like structure, suggesting a low likelihood of association with the biopsy. It was considered to be caused by tumor progression and thinning of the diverticular wall.

Complete surgical resection remains the standard treatment for localized GISTs. Minimally invasive approaches are recommended in current guidelines for tumors 5 cm or smaller.^7)^ Although large tumors have traditionally posed technical challenges, recent advances in minimally invasive surgical techniques have demonstrated the feasibility and safety of laparoscopic resection, even for tumors exceeding 10 cm.^8)^ All previously reported cases of jejunal diverticulum-like GISTs were treated via open surgery; however, in our case, laparoscopic resection was successfully achieved with negative surgical margins, even in an emergency setting. Given the patient’s history of sarcoidosis and ongoing corticosteroid therapy (prednisolone 10 mg/day), which increased the risk of surgical site infection and wound complications, a laparoscopic approach was selected to minimize wound size. Furthermore, an intracorporeal anastomosis of the transverse colon was performed to reduce the extent of colonic mobilization.

Risk stratification systems such as the modified Fletcher and Miettinen/the Armed Forces Institute of Pathology (AFIP) classifications incorporate tumor size, mitotic index, and primary tumor site.^7)^ In the present case, the tumor measured 10.2 cm and originated from the jejunum, which placed it in the high-risk category despite a relatively low mitotic index (2 per 50 HPF). High-risk GISTs have a well-documented tendency toward early recurrence—particularly hepatic metastasis—and the appearance of liver metastases 9 months postoperatively is consistent with the expected clinical course of this risk group.

For high-risk GISTs, adjuvant imatinib is strongly recommended to reduce recurrence risk. However, in this patient, initiation of therapy was delayed because of underlying cardiac sarcoidosis, a condition in which tyrosine kinase inhibitors may exacerbate cardiotoxicity.^9)^ This situation highlights a complex therapeutic dilemma: balancing the clear oncologic benefit of early adjuvant imatinib against the potential for serious cardiac complications. The subsequent development of liver metastases underscores the clinical consequences of delayed therapy and reinforces the importance of individualized multidisciplinary decision-making for patients with significant comorbidities.

An important consideration is whether the minimally invasive approach may have influenced these oncological outcomes. In this case, however, the perforation had already resulted in diffuse intraperitoneal contamination before surgery. Thus, the biological event most relevant to recurrence risk was the preoperative perforation itself. Although laparoscopic surgery cannot reverse the oncologic impact of perforation, negative margins were secured in our case, and there was no additional intraoperative tumor manipulation beyond what had already occurred due to perforation. Therefore, the early hepatic metastases are more plausibly attributed to the intrinsic high-risk biology and the perforated presentation rather than to the choice of surgical approach.

Careful consideration of indications is essential when performing laparoscopic surgery safely for perforated jejunal tumors with peritonitis. At a minimum, the patient’s vital signs must be stable enough to tolerate pneumoperitoneum. In addition, it is desirable to have an experienced surgical team—including the operating surgeon, assistant, scrub nurse, and anesthesiologist—and a facility capable of providing comprehensive postoperative management such as intensive care. During the operation, it is also crucial to prioritize patient safety and to convert to open surgery without hesitation if necessary.

Several mechanisms have been proposed for jejunal GISTs with a diverticulum-like appearance, and establishing a definitive diagnosis is often challenging.^3)^ A case of GIST arising within a preexisting true jejunal diverticulum has been previously reported.^10)^ However, in the present case, the diverticular wall was completely replaced by tumor cells, and there was no histological evidence suggesting the preexistence of a true jejunal diverticulum. Thus, it is likely that the diverticulum-like structure developed as a pseudodiverticulum in accordance with the outward expansion of the GIST originating within the jejunal wall. GISTs are known to grow exophytically,^11)^ and those arising in the jejunum have been reported to proliferate by replacing the bowel wall.^3)^ Furthermore, when a gut-replacing type of small intestinal GIST develops a diverticulum-like structure, it is often accompanied by perforation due to the fragility of the bowel wall, which is consistent with the clinical course in the present case.^3)^

## CONCLUSIONS

In conclusion, although jejunal diverticulum-like GISTs are difficult to diagnose and are often both large and perforated, this case demonstrates that laparoscopic resection is a feasible and safe treatment option, even for rare and complex presentations.

## DECLARATIONS

### Availability of data and materials

The data supporting the findings of this study are available from the corresponding author upon reasonable request.
